# Evaluation of combined high-efficiency DNA extraction and real-time PCR for detection of *Mycobacterium avium* subsp. *paratuberculosis* in subclinically infected dairy cattle: comparison with faecal culture, milk real-time PCR and milk ELISA

**DOI:** 10.1186/1746-6148-8-49

**Published:** 2012-05-02

**Authors:** Katarina Logar, Rok Kopinč, Petra Bandelj, Jože Starič, Aleš Lapanje, Matjaž Ocepek

**Affiliations:** 1 Veterinary Faculty, Institute of Microbiology and Parasitology, Gerbičeva 60, Ljubljana, 1115, Slovenia; 2 Institute of Physical Biology, Toplarniška 19, Ljubljana, 1000, Slovenia; 3 Veterinary Faculty, Clinic for Ruminants, Gerbičeva 60, Ljubljana, 1115, Slovenia

## Abstract

**Background:**

Johne’s disease is caused by *Mycobacterium avium* subsp. *paratuberculosis* (Map) and it is one of the most important diseases in cattle worldwide. Several laboratory tests for Map detection are available; however, these are limited by inadequate sensitivity and specificity when used in subclinically infected populations. To identify Map shedders in subclinically infected cattle, we used a new, high-yield method for DNA-extraction from Map in faeces combined with quantitative real-time PCR (qPCR) for amplification of the insertion sequence IS*900* of Map (HYDEqPCR). Evaluation of HYDEqPCR was carried out in comparison with faecal culture, milk qPCR, and milk enzyme-linked immunosorbent assay (ELISA), on 141 faecal and 91 milk samples, from 141 subclinically infected dairy cattle.

**Results:**

The qPCR proved to be highly sensitive, with a detection limit of 2 IS*900* DNA copies/μl in 67 % of the reactions. It also showed 100 % specificity, as determined from 50 Map and non-Map strains, and by the sequencing of qPCR amplicons. The detection limit of HYDEqPCR was 90 Map/g Map-spiked faeces, which corresponds to 2.4 colony forming units/g Map-spiked faeces, with an estimated efficiency of 85 % (±21 %). When tested on the field samples, HYDEqPCR showed 89 % of the samples as positive for Map, whereas faecal culture, milk qPCR, and milk ELISA detected 19 %, 36 % and 1 %, respectively. Fisher’s exact tests only show statistical significance (*p* ≤0.05) for the correlation between HYDEqPCR and faecal culture. The agreement between HYDEqPCR and milk qPCR and milk ELISA was poor, slight, and non-significant.

**Conclusions:**

This study highlights the advantages of HYDEqPCR for detection of Map in subclinically infected populations, in comparison with faecal culture, milk qPCR and milk ELISA. HYDEqPCR can detect low-level Map shedders that go undetected using these other methods, which will thus underestimate the proportions of Map-shedders in herds. Identification of these shedding animals is extremely important for prevention of the spread of Map infection in an animal population. Due to the relatively high sensitivity and specificity of HYDEqPCR, it can be applied to test for Map at the herd or individual level, regardless of animal age or production stage. HYDEqPCR will allow early detection and control of Map in any population at risk.

## Background

Johne’s disease, or paratuberculosis, is a chronic granulomatous enteritis that predominantly affects ruminants. It is caused by *Mycobacterium avium* subsp. *paratuberculosis* (Map). Infections with Map in bovine herds result in significant economic loss in the dairy industry, through slow progressive wasting and the subsequent death of the infected animals [1]. However, although Map-infected animals might not show symptoms of Johne’s disease for two to 10 years [2], during this time they shed Map into the environment. Identification of subclinically infected animals is therefore important, as they are the main cause of maintained infection of a herd and of transmission of Map infections between herds and premises [3]. The resulting continual exposure of susceptible herd-mates to environments containing Map-contaminated faeces (e.g. in the soil, water and feed) is the most common source of infection, and ultimately results in new infections [4]. To limit the spread of Johne’s disease, prompt measures need to be taken to reduce the risk of new infections, and thus early detection of subclinically Map-infected shedders is essential. This, however, is hampered by the lack of sufficiently sensitive, specific, reliable and fast laboratory tests [4].

The ‘gold standard’ for Map identification is still based on bacterial culture on solid media of faecal samples [4]. The slow growth (up to 16 weeks) and false negatives in samples that have low concentrations of Map makes it difficult to implement efficient protective strategies in an animal population when the Map identification is solely based on bacterial cultivation [5].

Likewise, enzyme-linked immunosorbent assays (ELISA) for the detection of antibodies against Map in milk and serum lack sensitivity. Generally, infected animals do not build up a measurable antibody titre until the later clinical stages of Johne’s disease. Therefore, ELISA is not suitable for detecting subclinically infected animals [5].

Quantitative real-time PCR (qPCR) is an alternative to bacterial culture and immunological methods. qPCR is a rapid test that can provide higher throughput and sensitivity [6-8]. However, a lack of high-yield extraction methods for mycobacterial DNA from complex matrices like faeces and milk still hinders the use of qPCR in diagnostics. Indeed, the high lipid content of the Map cell wall renders these bacteria inherently difficult to be lysed/ destroyed, which thus results in low DNA recovery, and further affects the sensitivity and efficiency of qPCR. In addition, complex biological matrices like faeces and milk contain a number of PCR-inhibiting substances (i.e. complex polysaccharides, polyphenols, bile salts, Ca^2+^ ions) that are difficult to eliminate using standard DNA extraction methods. An important property of an efficient DNA extraction method is thus also to remove these inhibitors.

Therefore, the aim of the present study was to detect subclinically Map-infected shedders (first and second stage) [2] based on an improved molecular detection method. We introduced a highly sensitive and specific, and high-efficiency, qPCR assay with the use of internal amplification control (IAC), to achieve the lowest possible limit of detection (LOD) of Map. This qPCR assay was further used to evaluate the efficiency of a newly developed DNA extraction kit that uses non-hazardous chemical and physical lysis for rapid and high-yield extraction of Map DNA from faeces.

To explore the usefulness of this new procedure of high-yield DNA extraction combined with qPCR (HYDEqPCR), this was applied to the detection of Map in samples from a farm with a subclinically infected cattle population and a previous history of Johne’s disease. The results are compared with those obtained using conventional bacterial culture, milk qPCR, and the milk commercial ELISA test. HYDEqPCR is presented as the method of choice for screening of Map in such populations, on a herd and on an individual basis.

## Results

### Performance of the IS*900* qPCR assay

**Figure 1:**
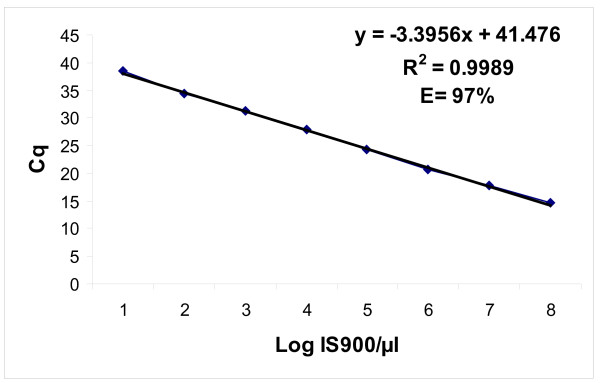
**Linear regression curve for the plasmid DNA.** The dilutions of the plasmid DNA (carrying the IS*900* DNA insert of the clinical Map isolate from the Internal Collection of the Veterinary Faculty, Institute of Microbiology and Parasitology, Ljubljana) in sterile distilled water are shown on a logarithmic scale, plotted against the corresponding Cq values.

When the plasmid carrying insertion sequence IS*900* was applied for the amplification, the linear regression of the qPCR assay resulted in an amplification efficiency of 97 % and a linear dynamic range spanning 8 log dilutions (quantification cycles [Cq] from 14.34 to 38.96), with an R^2^ of 0.9989 (Figure 1). The limit of quantitation (LOQ) was as low as 20 IS*900* copies per reaction (mean Cq, 38.43 ±0.31). The LOD was determined as 2 IS*900* copies, which were detected in 4 of 6 reactions. As expected, the highest variation of Cq within a triplicate was at the highest dilution (Cq, 39.63 to 41.14), due to stochastic effects that typically manifest at concentrations of ≤10 target copies per reaction [9]. A sample was considered to be Map positive when an amplification threshold was reached before a Cq value of 41.14, as this was the LOD of the qPCR, determined with a specific IS*900*-containing plasmid standard, with the signal output typical of an amplification curve.

The analytical specificity with the tested reference strains, the clinical Map isolates, and the non-Map strains was 100 %. The signal of the internal amplification control was positive in all of the reactions. All of the sequenced qPCR amplicons were also 100 % specific for Map, as seen after a comparison using BLAST of the sequences in publicly available sequence databases for similarity-based species identification.

### Evaluation of the DNA isolation procedure from faeces

**Figure 2:**
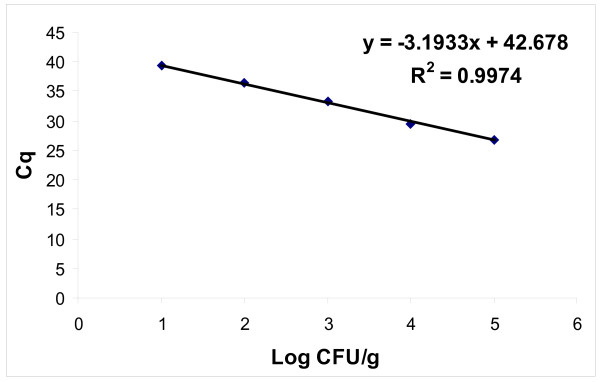
**Quantitative detection of*Mycobacterium avium*subsp.*paratuberculosis*in Map-spiked faeces using HYDEqPCR.** Linear regression curve of the isolated Map DNA plotted against the logarithm of the Map CFU/g faeces determined.

To evaluate the isolation procedure, the yield of IS*900* DNA copies from Map isolated from Map-spiked faecal samples was analysed. The IS*900* DNA isolation efficiency ranged from 65 % ±38 % to 112 % ±30 % of the input of serial-diluted bacterial suspensions of Map cells (Table 1). Experimentally, we detected 90 Map cells/g Map-spiked faeces according to the detected number of IS*900* copies in 5 of 6 reactions, taking into consideration that there are on average 15 IS*900* copies per single Map cell [10]. The LOD was equivalent to 2.4 colony forming units (CFU)/g Map-spiked faeces (Figure 2). All of the negative isolation extraction controls were negative for Map. There was also no significant qPCR inhibition by the sample matrix, as seen by a comparison of the Cq values for the IAC in samples and in sterile distilled water. The differences between the variances of the Cq for the IAC in the sample matrix and sterile distilled water were not statistically significant (*p* ≥0.05).

**Table 1:** **Efficiencies of the total*Mycobacterium avium*subsp.*paratuberculosis*DNA isolation procedure from the faeces**

**Theoretical input of Map cells per g faeces**^**a**^	**Experimental mean output of Map cells per g faeces**^**b**^	**Mean DNA isolation efficiency (%)**^**c**^
900,000	586,667	65 ±38
90,000	82,833	92 ±16
9,000	6,747	76 ±3
900	704	78 ±19
90	100	112 ±30

Map, *Mycobacterium avium* subsp. *paratuberculosis.*

^a^Determined by OD_600_ of bacterial suspension.

^b^Calculated as the mean of the recovered IS*900* copy number from IS*900* qPCR divided by 15, considering there is on average 15 IS*900* copies per single Map cell.

^c^Calculated as the mean yield obtained using HYDEqPCR divided by the theoretical number of Map cells added into the faeces, multiplied by 100.

### Detection of Map in faecal and milk samples

**Figure 3:**
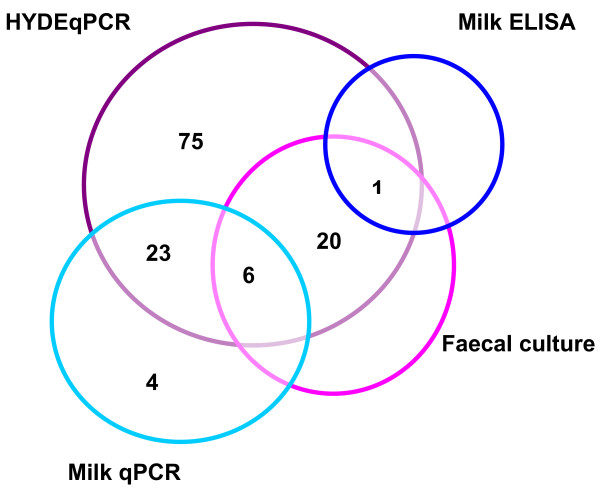
Identification of the Map-positive faecal and milk samples, as detected using the four detection methods.

All of the positive faecal and milk samples are summarised in Table 2. The association between the positive samples detected by the four methods of HYDEqPCR, faecal culture, milk qPCR and milk ELISA are presented in Figure 3. None of the animals was confirmed positive across all four of these methods, and none of the negative extraction controls were seen as positive.

**Table 2:** **Proportion of*Mycobacterium avium*subsp.*paratuberculosis*positive faecal and milk samples according to the detection methods**

**Animal age group (n)**	**HYDEqPCR**	**Faecal culture**	**Milk qPCR**	**Milk ELISA**
4-10 years (46)	40/46 (87 %)	6/46 (13 %)	18/46 (39 %)	0/46 (0 %)
2-4 years (75)	67/75 (89 %)	15/75 (20 %)	15/45 (33 %)	1/45 (2 %)
≤6 months (20)	18/20 (90 %)	6/20 (30 %)	/	/
**All animals** (141)	125/141 (89 %)	27/141 (19 %)	33/91 (36 %)	1/91 (1 %)

n: number of animals.

Of the 27 animals that were positive by culture, 22 (82 %) were low shedders, 3 (11 %) moderate shedders, and 2 (7 %) heavy shedders, as determined from the colony counts. The mean Cq of the HYDEqPCR was 36.66, 35.11, 26.44 for the low, moderate and heavy shedders, respectively, which corresponds to 19 CFU/g faeces, 56 CFU/g faeces and 28,848 CFU/g faeces, respectively. One of the heavy shedders was classified as a ‘super-shedder’, as it was shedding more than 10,000 Map CFU/g faeces according to faecal culture (145,518 Map CFU/g faeces as evaluated by HYDEqPCR) [11]. There were no statistical differences (*p* ≥0.05) for the average Cq values per PCR reaction in terms of the age groups of the animals.

### Correlations between the results of the Map detection methods

The kappa coefficients for agreement of the results across test pairs of the four Map detection methods ranged from −0.022 to 0.107. The only statistically significant kappa 12coefficients were for the test pairs between milk ELISA and faecal culture (*p* = 0.024), and between faecal culture and HYDEqPCR (*p* = 0.039); their corresponding kappa values can be interpreted as showing slight agreement between these test results. The other four kappa coefficients were highly insignificant (*p* ≥0.05) with poor and slight agreement seen (Table 3). For the two test pairs with statistically significant kappa values, the Fisher’s exact test was applied to test the null hypothesis of no association between the outcomes. The test pair of faecal culture and milk ELISA indeed showed no significant association (*p* = 0.165), while, in contrast, the test pair of HYDEqPCR and faecal culture showed significance (*p* = 0.027).

**Table 3:** Results for the chi-squared test and kappa coefficients for the four Map detection methods

	**HYDEqPCR**	**Faecal culture**	**Milk ELISA**
**HYDEqPCR**			
N		141	
chi-squared		4.275	
P		0.039	
Kappa		0.059	
**Milk qPCR**			
N	91	91	91
chi-squared	0.289	0.841	0.575
P	0.591	0.359	0.448
Kappa	-0.027	0.084	-0.022
**Milk ELISA**			
N	91	91	
chi-squared	0.111	5.123	
P	0.739	0.024	
Kappa	0.002	0.107	

n: number of samples.

*p* ≤0.05 indicates significant association between test results.

Kappa coefficient value of 1.00 indicates perfect agreement and a value of 0.00 indicates that any agreement is no better than chance.

## Discussion

Map infection of domestic-food-producing animals is associated with significant economic loss to the livestock industry worldwide. At present, preventive strategies to restrict the spread of Map in animal populations and to limit the economic loss are not satisfactory. This is because of the relatively low sensitivities of the currently available tests, which fail to detect many subclinically (first and second stage) Map-infected animals [11].

The present study describes an improved method for the detection of Map DNA in bovine faeces. Comparisons between the results of this new HYDEqPCR procedure and the other Map detection methods that are mainly used routinely by veterinary diagnostic laboratories (faecal culture, milk ELISA and milk qPCR) show that the proportion of Map shedders in an infected herd can be greatly underestimated. The use of this new HYDEqPCR procedure to efficiently and rapidly detect Map shedders will thus allow improved herd management for reduced Map transmission among animals.

To our knowledge, the detection limit of HYDEqPCR of 2.4 CFU/g Map-spiked faeces and/or 90 Map/g Map-spiked faeces are the lowest LOD values for Map that have been published to date. Considering Map-spiked faeces, three studies reported LOD values of 1,100 Map/g [7], 250 Map/g [12] and 3 CFU/g [13].

HYDEqPCR proved to be efficient in terms of DNA yield and purity, with dilution of the isolated DNA from the faecal samples not necessary, as our tests indicate that the sample matrix did not contain any inhibitory activities. With the use of the IAC in a qPCR assay, it is possible to distinguish between the false-negative and true-negative Map results. The mean efficiency of this DNA Map isolation procedure from faeces was 85 % (±21 %), which is better than has been previously reported [7]. The primer set [14] and the probe for the IS*900* qPCR were specifically selected to avoid IS*900*-like sequences that can be recognised in some other mycobacteria [15-17]. Despite the concerns over the specificity of this IS*900* region, and hence possible false-positive results due to other IS*900*-like sequences, we selected it as the qPCR target. Indeed, many alternative targets (f57, HspX, genes 251 and 255, ISMav2, ISMpa1, ISMAP02) have been used for the detection of Map, and although f57, HspX and ISMAP02 are unique to Map, they are present in a single copy and six copies, respectively in the Map genome [18]. Thus, IS*900* remains one of the preferred target sequences for amplification of Map-specific loci, as its multi-copy presence (14–18 copies) [10] in the Map genome can provide for much more sensitive real-time PCR assays compared to assays searching for a single to six copies gene targets [19]. In this case, many of the samples with low numbers of Map will still not be detected.

These limitations in sensitivity of the available diagnostic tests for the detection of Map infections at subclinical stages was one of the challenges that motivated the present study. Moreover, the studies that have reported that IS*900*-like sequences are the reason for false positive results have used conventional PCR assays [15-17], where it is known that the specificity is lower than that of real-time PCR assays. Furthermore, we took all of the necessary steps to avoid false-positive results in the present study. Thus, through our combination of the sequencing of the qPCR amplicons and all of our other qPCR specificity confirmation, we have been able to exclude false-positive results.

The results of HYDEqPCR were here compared with those of conventional faecal culture, milk qPCR, and commercial milk ELISA. The proportions of Map-positive samples from all of the animals identified with HYDEqPCR, faecal culture, milk qPCR and milk ELISA were 89 %, 19 %, 36 % and 1 %, respectively. This is in agreement with the results of a previously published study, where again, faecal PCR performed better than faecal culture and milk PCR [20]. However, the data in the present study are contrary to a report that showed that more positive results were detected by milk ELISA, followed by faecal culture and then faecal PCR [21]. However, a certain degree of discrepancy between studies must be expected, due to the differences in the protocols that are used across different laboratories. These differences can arise from the variable efficiencies of DNA extraction and purification procedures for the subsequent molecular testing, the media used, the preparation of the inoculum for culturing, and the various sensitivities and specificities of the molecular, ELISA and culture tests. Discrepancies between the results of different studies can also be affected by the selection of the animals tested, in terms of their ages and their Map infection stage, as well as of the overall herd prevalence.

Nevertheless, HYDEqPCR detected 70 % (98/141) positive samples in those that were deemed negative by bacterial culture. Based on these results of the bacterial cultures and the corresponding mean Cq values of HYDEqPCR, we can clearly distinguish between heavy and low-to-moderate shedders of Map. However, we cannot distinguish between low and moderate faecal shedders of Map. The lack of agreement between the low and moderate shedding as determined by faecal culture and the Cq values of HYDEqPCR casts doubt on these specific definitions of low and moderate shedding as determined by faecal culture. The proportion of low and moderate shedders as determined by faecal culture might indeed be underestimated, because a significant fraction of the Map can be killed during the decontamination procedure or because the clumping of several bacteria might form a single colony. Also, not all of the Map that are present in faeces can be cultured. On the other hand, the qPCR results can overestimate the viable Map concentration, as the non-viable bacteria are also determined with this method. Considering these positive and negative aspects of both of these diagnostic methods, there is now the argument that real-time PCR should become the gold standard instead of faecal culture, as over more recent years, various studies have shown better sensitivity and specificity for real-time PCR compared to bacterial culture [22].

In the present study, the high Cq values per PCR reaction across all of the age groups of the animals indicated low concentrations of Map in the faeces samples, which might also have been a reason for the relatively bad performance of the bacterial culture method. It has previously been reported that the sensitivity of bacterial culture often depends on the stage of the infection. The cows in the first stages of infection are known to shed low levels of Map in their faeces intermittently, and thus only 15 % to 20 % of these animals can be detected by faecal culture following a single test [2]. The results of the highly sensitive HYDEqPCR suggest that the low-level Map shedders that were detected were either passive shedders (the so-called ‘pass-through’ of Map in the intestinal tract) due to oral consumption of Map or were actively infected (most likely in the first and second stages of infection). This is further confirmed by the good ‘body condition score’ of the animals and the absence of clinical symptoms that are characteristic of Johne’s disease. Moreover, a super shedder was identified within the tested herd. It is known that such super-shedder cows will not have clinical signs initially, and so they represent a major concern for the spread of Map to other cows. Previous studies in dairy herds have reported 40 % to 60 % as low shedders in herds [23]. For many of the animals, this might reflect passive shedding after the consumption of water and feed that is contaminated through the presence of a small number of super shedders [11]. Indeed, as little as 1 g manure from a super-shedder can result in passive shedding of Map in an uninfected cow [11,24]. It has also been reported that low-level Map shedders are more likely to be truly infected with Map, than to just be undergoing passive shedding [25]. However, whether cows are passive shedders or are truly infected cannot be differentiated *ante-mortem*. Despite this, the indication is that the incidence of subclinically manifested paratuberculosis is underestimated at present.

This high percentage of Map positivity in faeces was definitely unexpected, and especially in the group of calves aged ≤6 months. However, these results strongly suggest that the majority of these animals, including the calves, must have been in contact with Map, which might be explained in terms of bad hygiene management practices on the farm studied. Calves are most susceptible to Map infection, and on the particular farm where the present study was carried out, they were not separated from infected, shedding adult cattle. This contact between the calves and adult cow faeces has been described as the most important risk factor of Map transmission to calves [26]. Calf to calf transmission [27] can also be one of the reasons explaining these high percentages of Map detection in the calf faecal samples. According to the results of hazards models on age at onset of faecal culture positivity based on results of individual culture for Map, the predicted proportion of cattle with an onset of shedding before 2 years of age is 30.3 % (95 % CI: 1.8 % - 91.8 %) in herds with an apparent prevalence ≥0.2 [28], and this is in agreement with the culture results of the present study. Moreover, *in-utero* infections cannot be excluded [29].

The milk qPCR here detected Map in 36 % (33/91) of the animals. The lower rate of positive samples detected by milk qPCR in comparison with HYDEqPCR were expected, as Map can be detected in milk only from animals in stages II-IV of Map infection [22]. Milk qPCR is, therefore, inadequate to investigate the incidence of infection in a population of animals or in an individual animal. However, when the Map distribution in milk is investigated, milk qPCR remains a useful tool to provide information relating to Map circulation in the food chain. Milk sampling is also more convenient, as the samples can be obtained non-invasively. Here, milk qPCR defined four animals as Map positive that were Map negative by HYDEqPCR and faecal culture. This divergence can be explained, however, as it is known that the shedding of Map in faeces and milk is not synchronised [30].

With milk ELISA, we detect only 1 % (1/91) of the animals as positive for Map. This might be due to the low levels of the antibodies in these subclinically infected animals, such that they are not generally detected by commercial ELISAs [22]. Indeed, the one animal that tested positive for this milk ELISA was recognised as a super shedder, as it had a very high concentration (3.2 × 10^6^) of Map cells per g faeces when determined by HYDEqPCR. This concentration was also the highest concentration detected within the herd in the present study. Therefore, milk ELISA appears to be an inappropriate method for the detection of subclinically infected animals, at least when carried out with the kit that was used in the present study.

Agreements between HYDEqPCR and faecal culture, milk qPCR and milk ELISA were also searched for. There was some agreement between HYDEqPCR and faecal culture. Similar agreement between these two methods has been seen previously, according to the Cohen’s kappa coefficients [20]. The low kappa coefficients in the present study for these two methods with relatively high specificities is an indicator of the higher sensitivity of HYDEqPCR when compared to faecal culture. The estimated agreements between HYDEqPCR and milk qPCR, and HYDEqPCR and milk ELISA were poor and slight, respectively, with no significance seen. Each of these methods detect different targets (bacterial DNA, antibodies), which have different disease dynamics and will thus be detected at different stages of a Map infection. The poor agreement between HYDEqPCR and milk qPCR might also be explained by the different timing of the shedding of Map in faeces and milk, as mentioned above.

## Conclusions

The present study highlights the advantages of HYDEqPCR over conventional faecal culture, milk qPCR and milk ELISA for the detection of Map in subclinically infected animal populations. HYDEqPCR detected low-level Map shedders in the herd that could not be detected by the other methods applied here. The results of the present study strongly suggest that the proportion of low-level Map shedders in an animal population will be underestimated at present. Shedding by these animals will mean continual exposure to a contaminated environment, which will ultimately result in Map infection of the healthy herd-mates. Therefore, the detection of Map shedders and the prevention of transmission of Map among animals and between herds or species is of great importance for the control of the spread of Map infection. The high efficiency of our DNA extraction technique, combined with the excellent sensitivity of qPCR, will also allow for pooled faecal sample testing, to determine whether a population/ farm/ barn is contaminated with Map. This can also be implemented in animals regardless of their ages and production stages, which will allow early detection and control of Map infections in any population at risk.

## Methods

### Animals and samples

Faecal and milk samples were collected from cows from a free-ranging dairy farm with a known history of Johne’s disease that is located close to the town of Postojna in the south-west of Slovenia. In total, 141 animals were included, which were divided into three age groups: 4–10 years (n = 46), 2–4 years (n = 75), which included 30 heifers, and calves aged ≤6 months (n = 20). The mean body condition score (according to US 0–5 scale [31]) of the adult animals were: aged 4–10 years, 2.96 ±0.47 (range, 2.25-4.5); and aged 2–4 years, 3.04 ±0.44 (range, 2.0-4.0). The body condition of the calves was appropriate. The tested population did not show any clinical symptoms that were characteristic of Johne’s disease.

A total of 141 faecal samples and 91 milk samples were collected. The faecal samples were collected from the animal rectum using a sterile glove and a rectal sleeve. The milk samples were collected in sterile 50-ml tubes after the initial four squirts were discarded. The samples were delivered to the laboratory within 1 h of their collection, stored at 4°C, and processed within 4 days of collection.

### Faecal culture

Ten grams of each faecal sample were placed into a sterile 50-ml tube containing 30 ml sterile distilled water, and then mixed for 30 min at 250 rpm. The solutions were then allowed to settle for 30 min. Five millilitre aliquots of each supernatant were removed and added to 25 ml 0.90 % hexadecylpyridinum chloride. The remainders of the samples were stored at −80°C for DNA extraction.

The supernatants and hexadecylpyridinum chloride were mixed for 30 min at 250 rpm, and then incubated at room temperature for 18 h. After this incubation, the samples were centrifuged for 20 min at 1,400× *g*, and the sediments were resuspended in 1 ml sterile distilled water. Then 200 μl from each of these resuspended samples was inoculated onto each of the Herrold’s egg yolk agar slants that were supplemented with mycobactin J, amphotericin B, nalidixic acid, and vancomycin (HEYA; Becton Dickinson, Sparks, USA). The slants were incubated at 37°C and the resultant colonies were counted weekly, over 16 weeks. The final results were evaluated as low (1–9 CFU/tube), moderate (10–50 CFU/tube) or heavy (>50 CFU/tube) faecal shedding [23]. The colonies were stained using the Ziehl-Neelsen method, and examined under light microscopy for the presence of acid-fast bacilli. The identities of all of the colonies were additionally confirmed by amplification of IS*900* by PCR, as described previously [32].

### Isolation of DNA from faeces

The DNA extraction from the faeces was performed using SmartHelix™ First DNAid kit (Institute of Physical Biology, Slovenia), according to the protocol supplied. The frozen samples were thawed, and 2 ml of each sample was centrifuged for 5 min at 10,000× *g*. The pellets were resuspended in lysis buffer with added proteinase K (Sigma-Aldrich, USA) and ≤106-μm-diameter glass beads (Sigma-Aldrich, USA). This was followed by bead beating on a MagNALyser instrument (Roche, Switzerland), at 6,400 rpm for 60 s, and an incubation at 56°C for 15 min. The bead beating and the incubation were repeated. This was followed by an additional bead beating step at 6,400 rpm for 60 s, after which the samples were boiled for 10 min and allowed to cool to room temperature. The samples were then further centrifuged at 10,000× *g* for 5 min, after which the supernatants were transferred to new tubes, and mixed with 1050 μl of binding buffer. The samples where then loaded onto DNAid spin columns and centrifuged at 16,000× *g* for 1 min, followed by rinsing of thecolumns two times with wash buffer with centrifugation at 16,000× *g* for 1 min and then finally for 5 min. The DNA was eluted from the DNAid spin columns into fresh tubes using 100 μl elution buffer and centrifugation at 16,000× *g* for 1 min. For each series of DNA extractions (21 samples), three negative extraction controls were processed simultaneously.

### Preparation of cloned IS*900* DNA

The qPCR amplification standard was prepared by cloning PCR-amplified IS*900* (with the same primers used for the Map detection) into a plasmid vector using the TOPO TA Cloning® kit (Invitrogen, USA). The isolated cloned IS*900* fragment was sequenced (Macrogen, The Netherlands), and thus confirmed to be 100 % specific for Map after comparison of the sequence with public available sequence databases for similarity-based species identification, using BLAST (http://blast.ncbi.nlm.nih.gov/Blast.cgi).

The copy numbers of the IS*900*-containing plasmid standard were determined by qPCR as follows. The pure vector plasmid pCR®2.1-TOPO® (Invitrogen, USA) was used as a quantification standard. For this purpose, we designed primers for the vector plasmid pCR®2.1-TOPO® (Invitrogen, USA) that target a region outside the insertion site, pCR21_2f (5’-TGCCTGCTTGCCGAATATC) and pCR21_2r (5’-CCACAGTCGATGAATCCAGAAA). The DNA concentration of the first dilution of pure pCR®2.1-TOPO® was measured using a Qubit 2.0 Fluorometer and Quant-iTdsDNA High Sensitivity Assay kit (Invitrogen, USA), and the IS*900*-containing plasmid copy numbers were calculated. Three serial 10-fold dilutions of the IS*900*-containing plasmid standard were run on a real-time PCR System 7500 (Applied Biosystems), along with six 10-fold serial dilutions of the pure vector plasmid pCR®2.1-TOPO®, all in triplicate. The 20 μl reactions contained 400 nM of each primer and 10 μl Power SYBR® Green PCR Master mix (Applied Biosystems, USA). The copy numbers of the IS*900*-containing plasmid were calculated using a standard curve constructed from 10-fold serial dilutions of pure pCR®2.1-TOPO®. The lowest dilution of the IS*900*-containing plasmid standard was selected and aliquoted for further qPCR Map-detection experiments. A separate aliquot was used in each quantification experiment.

### Set-up and evaluation of IS*900* qPCR assay

The qPCR was performed on a Light Cycler 2.0 real-time PCR machine (Roche Diagnostics, Switzerland). The oligonucleotide primers (TJ50, TJ51) [14], which form an amplicon of 136 bp, and a TaqMan probe 5’-6FAM-AAGACCGACGCCAAAGACGCTGCGA-BBQ-para TM (TIB Molbiol, Germany) targeting the IS*900* sequence (GenBank accession no. AJ250018 or/and X16293) were positioned in the Map-specific part of IS*900*. They were tested *in silico* using BLAST to confirm the Map specificity. The optimised reaction mixture contained: 12.5 μl 2× Maxima Probe qPCR Master Mix (Fermentas, Lithuania), 0.3 μM of each primer, 0.4 μM of the IS*900* TaqMan probe, 2.5 μl 10× Exo IPC mix containing the primers and the VIC-labelled probe (Applied Biosystems, USA), 0.5 μl 50× Exo IPC DNA (Applied Biosystems, USA), which was diluted 1:170, 5 μl of the DNA sample, and sterile distilled water to a final volume of 25 μl. The TaqMan® exogenous internal positive control (Applied Biosystems, USA) was included to monitor for any inhibition of the amplification reaction. The thermal cycling conditions were as follows: initial denaturation at 95°C for 10 min, followed by 50 cycles at 95°C for 15 s (denaturation), 58°C for 30 s (annealing), and 72°C for 30 s (extension). The final cooling step was for 30 s at 40°C. The Cq values were obtained using the Light Cycler 2.0 software, version 4.1.1.21 (Roche Diagnostics, Switzerland). Acquisition of the fluorescence signal (FAM, 530 nm; VIC, 560 nm) was performed during the annealing step. A colour compensation experiment was performed according to the manufacturer instructions (Roche Diagnostics, Switzerland). For all of the analyses, the automated method (F” max) was used. A non-template control (sterile distilled water) was used to monitor for potential contamination within the qPCR reagents.

The analytical specificity of the qPCR described was tested on 50 Map and non-Map strains (including official reference strains and clinical strains, as listed in file Additional file 1). The non-Map strains were selected based on their close genetic relationship to Map, their presence in the normal intestinal microflora of cattle, or their *in-vivo* growth similarities. We also selected 10 qPCR amplicons from naturally infected faecal samples from the investigated farm with Cq values ranging from 23.84 to 41.11 for sequencing (Macrogen, The Netherlands) to further confirm the specificity of the qPCR.

We determined the amplification efficiency, linear dynamic range, LOD and LOQ of the IS*900* qPCR assay. Ten-fold serial dilutions (from undiluted to 10^-9^ dilution) of the prepared IS*900*-containing plasmid standard were run in triplicate in two independent experiments. The calculated copy numbers of the IS*900*-containing plasmid standard were used for generation of a standard curve. The LOD was defined as the lowest concentration of the IS*900*-containing plasmid standard that gave a detected signal in at least one of three parallel reactions for each qPCR run. LOQ was defined as the lowest dilution at which all three parallel reactions were positive.

### Preparation of Map-spiked faeces and evaluation of the isolation procedure

Serial dilutions of Map cells were prepared for the spiking of faeces. Briefly, the Map strain from Internal Collection of the Veterinary Faculty, Institute of Microbiology and Parasitology, Ljubljana (previously isolated from a cow on the studied farm) was confirmed to be a cattle type strain [33], and was grown on Herrold’s egg yolk agar slants supplemented with mycobactin J, amphotericin B, nalidixic acid and vancomycin (Becton Dickinson, Sparks, USA). The bacteria were harvested and resuspended in phosphate-buffered saline (pH 7.4) using a sterile inoculating loop. Sterile glass beads (diameter, *ca*. 5 mm) (Assistant, Germany) were added, and the Map suspension was vortexed for 45 s to break down the clumps. The concentration of the stock suspension was estimated by comparing the optical density at 600 nm (OD_600_) with McFarland standards, which was further confirmed by counting the cells under light microscopy, using a Neubauer chamber under phase contrast. Estimation of the CFUs was carried out by plating serial dilutions (10^6^ to 10) in phosphate-buffered saline on Middlebrook 7 H10-OADC agar supplemented with mycobactin J (Becton Dickinson, Sparks, USA). Glass Petri dishes with agar of at least 5 mm thickness were sealed with Duraseal™ (Sigma-Aldrich, USA), put into polyvinyl bags, and incubated at 37°C for up to 12 weeks, at which time the colonies were quantified visually. An OD_600_ of 0.1 corresponded theoretically to 150 × 10^6^ bacteria/ml, while the estimations from the CFU plate counting resulted in approximately 8 × 10^6^ CFU/ml for the same OD_600_.

Serial dilutions of the stock suspension were used for spiking 10 g of faecal samples from a known Map-free donor cow, in 30 ml sterile distilled water. Two sets of spiked faeces were prepared for the DNA isolation, to enable testing in duplicate. Two negative sample controls were included to monitor for potential contamination during the isolation process. After DNA isolation, the efficiency of the isolation procedure was determined by IS*900* qPCR in two independent experiments. The LOD was determined as the lowest theoretical amount of Map cells per gram of faeces that resulted in a positive qPCR amplification signal in at least one of three parallel reactions for each qPCR run. The number of Map cells was calculated from the recovered IS*900* copy number divided by 15, considering there are on average 15 IS*900* copies per single Map cell [10]. The mean DNA isolation efficiency was calculated for each dilution, as the quotient of the recovered amount of Map cells and the theoretical input. To monitor for potential inhibition of the sample matrix, the IAC was amplified in sterile distilled water and sample matrix. Levene’s test for equality of variances was used to test for differences between the variances of the Cq for the IAC.

### Detection of Map DNA and anti-Map antibodies in milk

Milk samples (50 ml) were pre-processed to concentrate Map [34], and DNA was isolated using QIAamp DNA Blood Mini kit (Qiagen GmbH, Germany), as described previously [35]. In each series of DNA isolations (21 samples), three negative extraction controls were processed. The isolated DNA was tested for IS*900* by qPCR, as described above. The samples were also tested for the presence of antibodies, using Pourquier ELISA Paratuberculosis kit (Institut Pourquier, France).

### Statistical analysis

Statistical analysis of the results of the four Map detection methods was carried out to evaluate the new procedure in comparison with other detection methods, and to determine any correlations between them. The choice in the selection of the best method for screening for Map in a population or at an individual level is important in the management of paratuberculosis.

Chi-squared tests and Cohen’s kappa coefficients were calculated, to determine the agreement between each pair of methods. The following ranges were considered for interpretation of the kappa coefficients [36]: poor agreement, <0.00; slight agreement, 0.00-0.20; fair agreement, 0.21-0.40; moderate agreement, 0.41-0.60; substantial agreement, 0.61-0.80; almost perfect agreement, 0.81-1.00. A value of 1.00 indicates perfect agreement. A value of 0.00 indicates that any agreement is no better than chance. Kappa coefficients can also be negative, which is a sign that the two observers agreed less than would be expected just by chance.

Pearson chi-squared tests were used to test for statistical significance of the kappa coefficients for each pair of tests. F0r the test of independence, a chi-squared probability of ≤0.05 is used for rejection of the null hypothesis that the row variable is independent of the column variable, which means that there is agreement between the pair of tests.

Where the resulting 2 × 2 contingency table was not balanced and had at least one expected cell count with less than 5 observations for both tests, then the Fisher’s exact test was calculated to determine whether there was any non-random association between each pair of test results. The Fisher's exact test is substantially more accurate for the evaluation of the significance level, especially with small numbers of observations. The data were analysed using SPSS Inc., version 18.0.

### Ethics Statement

This study was performed with the approval of the Veterinary Administration of the Republic of Slovenia (VARS; consent reference 34401-25/2010/3). Regarding the statement of the VARS, which issues permission for studies on animals in the Republic of Slovenia, the non-invasive procedures (including sampling of animal faeces and milk) are not required to be approved by the Ethics Committee. Informed consent for the present study was also obtained from the farm owner.

## Authors’ contributions

KA designed the study, carried out the laboratory work, compiled and analysed the data, and drafted the manuscript. RK carried out part of the laboratory work (“Preparation of cloned IS*900* DNA”). PB carried out the sampling and helped in the laboratory work. JS coordinated and carried out the sampling and assessed the body condition scores of the animals. AL developed the SmartHelix™ First DNAid kit. MO helped to design the study, supervised the study, and coordinated and carried out the sampling. All of the authors have critically revised and approved the final manuscript.

## Supplementary Material

Additional file 1 Bacterial strains used to test the specificity of the IS*900* quantitative real-time PCR. The reference and clinical Map and non-Map strains used to test the specificity of the IS*900* quantitative real-time PCR are listed in the file. Click here for file
