# Supplementary material for: Evaluation of combined high-efficiency DNA extraction and real-time PCR for detection of Mycobacterium avium subsp. paratuberculosis in subclinically infected dairy cattle: comparison with faecal culture, milk real-time PCR and milk ELISA
Source: BMC Vet Res. 2012 May 2;8:49. doi: 10.1186/1746-6148-8-49 (PMC3423054; doi:10.1186/1746-6148-8-49)
Supplement: Additional file 1 — Bacterial strains used to test the specificity of the IS900 quantitative real-time PCR. The reference and clinical Map and non-Map strains used to test the specificity of the IS900 quantitative real-time PCR are listed in the file. [file 1746-6148-8-49-S1.pdf]

Additional Material

Bacterial strains used to test the specificity of the IS900 quantitative real-time PCR

a. Reference mycobacterial strains (n = 18)

	**Species**	**Source**
1	*M. terrae*	ATCC 15755
2	*M. tuberculosis*	ATCC 25177
3	*M. avium subsp. avium*	ATCC 25291
4	*M. intracellulare*	ATCC 35767
5	*M. bovis*	ATCC 19210
6	*M. chelonae*	ATCC 35752
7	*M. flavescens*	ATCC 14474
8	*M. malmoense*	ATCC 29571
9	*M. abscessus*	ATCC 19977
10	*M. scrofulaceum*	ATCC 1998
11	*M. szulgai*	ATCC 35799
12	*M. kansasii*	ATCC 12248
13	*M. marinum*	ATCC 927
14	*M. gordonae*	ATCC 14470
15	*M. xenopi*	ATCC 19250
16	*M. triviale*	ATCC 23292
17	*M. avium* subsp. *paratuberculosis*	ATCC 43015
18	*M. avium* subsp. *paratuberculosis*	ATCC 19698

ATCC, American Type Culture Collection

b. Clinical mycobacterial strains (n = 17)

	Species	Source
1	*M. celatum*	Deer (*Dama dama*), ICVF
2	*M. fortuitum*	Fish (*Xiaphophorus helleri*), ICVF
3	*M. marinum*	Fish (*Xiaphophorus maculatus*), ICVF
4	*M. marinum*	Fish (*Polcilia reticulate*), ICFV
5	*M. chelonae*	Fish (*Xiaphophorus helleri*), ICVF
6	*M. caprae*	Cattle (*Bos primigenius*), ICVF
7	*M. smegmatis*	VLA
8	*M. avium* subsp. *avium*	Pig (*Sus scrofa domesticus*), ICVF
9	*M. avium* subsp. *avium*	Pig (*Sus scrofa domesticus*), ICVF
10	*M. avium* subsp. *avium*	Hen (*Gallus gallus domesticus*), ICVF
11	*M. avium* subsp. *avium*	Hen (*Gallus gallus domesticus*, ICVF
12	*M. xenopi*	ICVF
13	*M. avium* subsp. *hominissuis*	Pig (*Sus scrofa domesticus*), ICVF
14	*M. avium* subsp. *paratuberculosis*	VLA
15	*M. avium* subsp. *paratuberculosis*	Cattle (*Bos primigenius*), ICVF
16	*M. avium* subsp. *paratuberculosis*	Cattle (*Bos primigenius*), ICVF
17	*M. avium* subsp. *paratuberculosis*	Cattle (*Bos primigenius*), ICVF

ICVF, Internal Collection of Veterinary Faculty, Ljubljana

VLA, Veterinary Laboratories Agency

c) Microorganisms other than mycobacteria (n = 15)

	Species	Source
1	*Clostridium botulinum* C	ICVF
2	*Clostridium perfringens* A	ATCC 25768
3	*Bacteroides fragilis*	ATCC 25285
4	*Campylobacter coli*	ICVF
5	*Campylobacter jejuni*	ATCC 33560
6	*Enterococcus faecalis*	ATCC 29212
7	*Escherichia coli*	ATCC 25922
8	*Escherichia coli* O157	ICVF
9	*Proteus mirabilis*	DSM 788
10	*Staphylococcus aureus*	ATCC 25923
11	*Salmonella* enteritidis	CAPM 5439
12	*Salmonella* typhimurium	ATCC 14028
13	*Clostridium difficile*	ATCC 9689
14	*Listeria innocua*	ATCC 33090
15	*Klebsiella pneumoniae*	ATCC 23357

ICVF, Internal Collection of Veterinary Faculty, Ljubljana

ATCC, American Type Culture Collection

DSM, Deutsche Sammlung von Mikroorganismen

CAPM, Collection of Animal Pathogenic Microorganisms
